# Selective Extraction of Lipophilic Bioactive Compounds from Industrial Root Meal of *Glycyrrhiza glabra* L.

**DOI:** 10.3390/molecules31142411

**Published:** 2026-07-08

**Authors:** Akbar Sanoev, Bakhodir Okhundedaev, Ildar Sham’yanov, Khayrulla Bobakulov, Sayyora Zaripova, Ruzali Botirov, Alimjan Sadikov, Shamansur Sagdullayev, Farida Ali, Eldar Garayev

**Affiliations:** 1 S.Y. Yunusov Institute of the Chemistry of Plant Substances, Academy of Sciences of Uzbekistan, Tashkent 100170, Uzbekistan; sanoev.a85@mail.ru (A.S.); boxundedayev@mail.ru (B.O.); i.shamyanov@mail.ru (I.S.); khayrulla@rambler.ru (K.B.); zaripovasayyora94@gmail.ru (S.Z.); botiroovr@mail.ru (R.B.); sadikov1948@bk.ru (A.S.); sh_sagdullayev@rambler.ru (S.S.); 2 Laboratory of Experimental and Technological, Azerbaijan Medical University, Baku AZ1022, Azerbaijan; feridenurane@gmail.com

**Keywords:** *Glycyrrhiza glabra* L., glabridin, triterpenoid aglycones, 3-oxoglycyrrhetinic acid, glycyrrhetinic acid, sequential extraction, bioactive compounds

## Abstract

Licorice (*Glycyrrhiza glabra* L.) root meal generated after industrial extraction of water-soluble constituents represents an underutilized secondary plant resource rich in lipophilic bioactive compounds. In this study, an efficient approach for the comprehensive recovery of hydrophobic biologically active substances from licorice root meal was developed. The method is based on sequential ethanol extraction followed by selective fractionation using a petroleum ether–ethyl acetate solvent system and chromatographic purification. As a result, a lipid fraction (1.1%) containing phytosterols (β-sitosterol and stigmasterol) was obtained, while the pharmacologically important isoflavan glabridin was isolated with a purity of 87.9% and a yield of 0.17%. In addition, triterpenoid aglycones, including 3-oxoglycyrrhetinic acid (0.39%) and glycyrrhetinic acid (0.21%), were successfully isolated and structurally confirmed by IR and NMR spectroscopy. Comparative solvent studies demonstrated that ethanol provides the highest extraction yield (7.1%) while maintaining high levels of glabridin and total flavonoids in the extracts. The results indicate that licorice root meal is a valuable secondary source of lipophilic bioactive compounds, and the proposed approach enables more efficient utilization of plant raw materials, reduction of industrial waste, and development of sustainable technologies for obtaining pharmacologically valuable compounds for pharmaceutical, cosmetic, and biomedical applications.

## 1. Introduction

*Glycyrrhiza glabra* L. (licorice) is one of the most valuable medicinal plants widely used in pharmaceutical, food, and cosmetic industries [1]. The plant is distributed throughout the Mediterranean region, the Middle East, Central Asia, and northwestern China. It is also widely found in Uzbekistan, where it forms extensive natural populations and represents an important source of medicinal and industrial raw materials [2].

The roots and rhizomes of *G. glabra* L. are characterized by a complex chemical composition containing numerous biologically active compounds belonging to different classes. Major groups of secondary metabolites include triterpenoids (up to 24%), represented mainly by glycyrrhizic acid, glycyrrhetinic acid and their derivatives; phenolic compounds (up to 6%), predominantly flavonoids including the pharmacologically important isoflavan glabridin; carbohydrates (up to 20%), mainly biologically active polysaccharides; lipids (up to 4%), including triglycerides, sterol esters, free fatty acids and phytosterols; as well as proteins (up to 10%) [1,2,3,4,5]. Among these compounds, glycyrrhizic acid and its aglycone glycyrrhetinic acid are of particular importance due to their pronounced anti-inflammatory, hepatoprotective, and antiviral properties, which explain their extensive application in medicine and food technology [6,7]. Flavonoids of licorice, especially glabridin, exhibit strong antioxidant, anti-inflammatory, and depigmenting activities and are widely used in pharmaceutical preparations and cosmetic formulations [8]. In addition, polysaccharides isolated from licorice roots possess immunomodulatory and antitumor activities [9,10], while the lipid fraction has been reported to exhibit anti-inflammatory and tissue-regenerating effects [11,12].

In recent years, considerable attention has been devoted to the development of sustainable technologies for the extraction, purification, and biomedical application of natural bioactive compounds. Advanced functional materials, green extraction strategies, and innovative approaches for the valorization of plant-derived substances have significantly expanded the possibilities for obtaining high-value products from renewable biological resources. Recent studies have emphasized the importance of environmentally friendly processing technologies, efficient utilization of plant residues, and integrated approaches for maximizing the recovery of biologically active compounds from medicinal plants [13,14,15,16,17,18].

Furthermore, the recovery of valuable phytochemicals from industrial plant-processing by-products has emerged as an important direction within the framework of circular bioeconomy and sustainable manufacturing. The utilization of secondary plant resources not only reduces industrial waste but also provides additional sources of pharmacologically relevant compounds, including flavonoids, triterpenoids, and other lipophilic constituents. These developments highlight the necessity of creating resource-efficient technologies for the comprehensive processing of medicinal plant materials and their industrial residues [19,20,21].

Industrial processing of licorice roots is mainly aimed at obtaining glycyrrhizic acid and related products from aqueous extracts [22,23]. Conventional technology involves multiple water extractions of the raw material at temperatures of 50–60 °C, followed by concentration of the combined extracts to obtain thick or dry extracts [24]. However, this process primarily targets water-soluble constituents and does not efficiently extract lipophilic compounds [25].

As a result, a considerable amount of secondary by-product, namely licorice root meal, is formed during industrial processing, accounting for approximately 30–60% of the initial raw material mass [26]. Although considered a waste product, this material still contains significant quantities of valuable compounds, including lipophilic constituents and phenolic compounds such as glabridin [27]. These findings indicate that licorice root meal is not a fully exhausted raw material and retains considerable chemical potential. One of the major limitations of conventional processing technologies is the incomplete recovery of hydrophobic biologically active compounds. During traditional aqueous extraction, lipids, glabridin, glycyrrhetinic acid, and its derivatives, including 3-oxoglycyrrhetinic acid, remain largely unextracted. The presence of these compounds in licorice root meal indicates the potential value of this secondary plant resource.

Despite the growing interest in the valorization of medicinal plant residues, information regarding the sequential recovery of lipophilic fractions, including glabridin, glycyrrhetinic acid, and 3-oxoglycyrrhetinic acid, from licorice root meal remains limited. Moreover, comprehensive approaches enabling the integrated utilization of this industrial by-product have not been sufficiently investigated.

Therefore, the development of scientifically grounded technologies for the deep processing of *G. glabra* L. root meal aimed at the comprehensive recovery of hydrophobic biologically active compounds represents an important task for improving the efficiency of plant raw material utilization and reducing industrial waste.

In this context, the aim of the present study was to develop a sequential extraction strategy for the comprehensive recovery of lipophilic biologically active compounds from *G. glabra* L. root meal and to evaluate the efficiency of the proposed approach for obtaining lipid fractions, glabridin, glycyrrhetinic acid, and 3-oxoglycyrrhetinic acid.

## 2. Results

Yield and purity of hydrophobic compounds isolated from the root meal of *G. glabra* L. are presented in Table 1.

### 2.1. Yield and Characterization of the Lipid Fraction

As a result of extraction, 11 g of a lipid fraction was obtained (1.1% of the air-dried root meal mass), representing a transparent oily liquid of yellowish color. Thin-layer chromatography (TLC) analysis confirmed the efficient extraction of lipid components from the root meal. Chromatographic separation of the lipid fraction allowed the isolation of two individual phytosterols.

**Figure 1:**
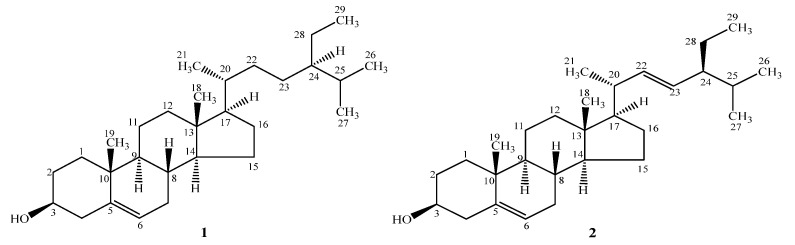
Chemical structures of β-sitosterol (**1**) and stigmasterol (**2**).

Chromatographic separation of the lipid fraction allowed the isolation of two individual phytosterols. According to spectral data, the isolated compounds were identified as β-sitosterol (**1**) and stigmasterol (**2**) (Figure 1).

β-Sitosterol (**1**). White crystals, melting point 140–142 °C. ^1^H NMR (400 MHz, CDCl_3_, δ, ppm, *J*/Hz): 3.52 (1H, *m*, H-3), 5.35 (1H, *d*, 3.6, H-6), 0.68 (3H, *s*, H-18), 1.01 (3H, *s*, H-19), 0.92 (3H, *d*, 6.2, H-21), 0.81 (3H, *d*,6.6, H-26), 0.83 (3H, *d*,5.2, H-27), 0.85 (3H, *t*,6.3, H-29). ^13^C NMR (100 MHz, CDCl_3_, δ, ppm): 37.4 (C-1), 31.8 (C-2), 71.9 (C-3), 42.4 (C-4), 140.9 (C-5), 121.8 (C-6), 32.1 (C-7), 32.0 (C-8), 50.2 (C-9), 36.3 (C-10), 21.2 (C-11), 39.9 (C-12), 42.4 (C-13), 56.9 (C-14), 24.4 (C-15), 28.4 (C-16), 56.2 (C-17), 12.0 (C-18), 19.5 (C-19), 36.6 (C-20), 18.9 (C-21), 34.1 (C-22), 26.2 (C-23), 45.9 (C-24), 29.8 (C-25), 19.2 (C-26), 19.9 (C-27), 23.2 (C-28), 12.3 (C-29).

Stigmasterol (**2**). White crystals, melting point 165–167 °C. ^1^H NMR (400 MHz, CDCl_3_, δ, ppm, *J*/Hz): 3.46 (1H, *m*, H-3), 5.29 (1H, *d*,5.3, H-6), 0.86 (3H, *s*, H-18), 0.94 (3H, *s*, H-19), 0.95 (3H, *d*,6.2, H-21), 0.84 (3H, *d*, 6.6, H-26), 0.77 (3H, *d*,5.2, H-27), 0.76 (3H, *t*, 6.3, H-29). ^13^C NMR (100 MHz, CDCl_3_, δ, ppm): 37.2 (C-1), 31.7 (C-2), 71.8 (C-3), 42.3 (C-4), 140.8 (C-5), 121.7 (C-6), 32.1 (C-7), 32.0 (C-8), 50.2 (C-9), 36.2 (C-10), 21.2 (C-11), 39.8 (C-12), 42.3 (C-13), 56.8 (C-14), 24.4 (C-15), 28.4 (C-16), 55.9 (C-17), 12.1 (C-18), 19.5 (C-19), 36.6 (C-20), 18.9 (C-21), 138.3 (C-22), 129.3 (C-23), 45.8 (C-24), 29.8 (C-25), 19.1 (C-26), 19.9 (C-27), 23.1 (C-28), 12.0 (C-29).

### 2.2. Isolation and Purification of Glabridin

Sequential extraction and chromatographic purification enabled the isolation of glabridin with progressively increasing purity. Initial column chromatography yielded a fraction containing 49% glabridin (5.7 g). Further chromatographic purification increased the glabridin content to 69.8% (2.8 g). Final purification on a Sephadex^®^ LH-20 column resulted in the isolation of 1.95 g of glabridin. HPLC analysis showed that the obtained product contained 87.9% glabridin.

The remaining 12.1% fraction is assumed to consist predominantly of structurally related flavonoids and minor phenolic constituents naturally occurring in licorice root meal. Although these accompanying compounds were not individually identified in the present study, their presence is consistent with the known phytochemical composition of *G. glabra* and the chromatographic characteristics of the isolated fraction. The obtained compound was a dark orange powder with a melting point of 236–238 °C.

**Figure 2:**
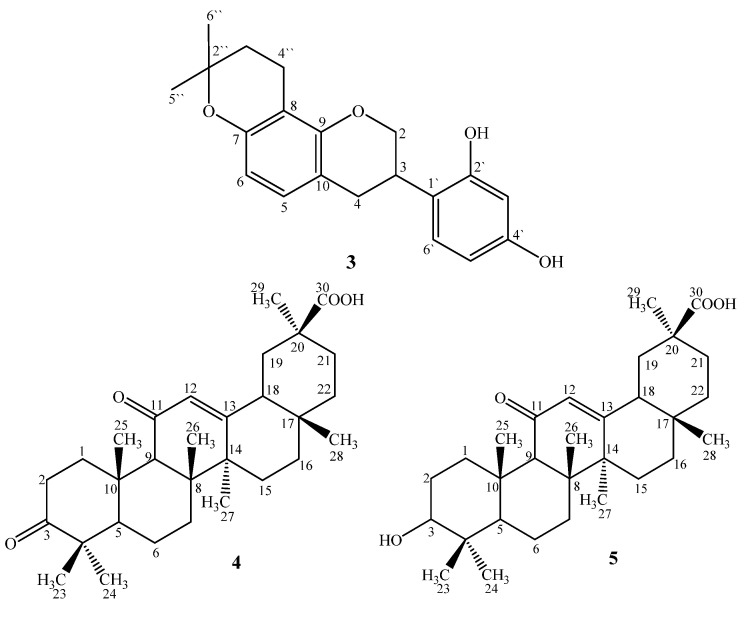
Chemical structures of glabridin (**3**), 3-oxoglycyrrhetinic acid (**4**) and glycyrrhetinic acid (**5**).

These ^1^H NMR spectral data correspond to glabridin (**3**) (Figure 2).

IR spectrum (ν, cm^−1^): 3528, 3351 (OH), 2965 (–CH_3_), 1607, 1519 (Ar), 1112, 1090 (C–O).

^1^H NMR (600 MHz, CDCl_3_, δ, ppm, *J*/Hz): 1.40 (3H, *s*, H-5″), 1.42 (3H, *s*, H-6″), 2.86 (1H, *ddd*, 15.7, 5.3, 1.8, H-4a), 2.95 (1H, *dd*, 15.6, 11.0, H-4b), 3.47 (1H, *m*, H-3), 4.00 (1H, *dd*, 10.2, 10.2, H-2a), 4.35 (1H, *m*, H-2b), 5.54 (1H, *d*, 9.6, H-3″), 6.30 (1H, *d*, 2.4, H-3′), 6.35 (1H, *d*, 8.26, H-6), 6.37 (1H, *dd*, 2.48, 8.28, H-5′), 6.63 (1H, *d*, 9.9, H-4″), 6.81 (1H, *d*, 8.11, H-5), 6.94 (1H, *d*, 8.35, H-6′).

The IR (Infrared spectroscopy) spectrum is characterized by absorption bands of hydroxyl groups (3528, 3351 cm^−1^), the aromatic system (1607, 1519 cm^−1^), and C–O bonds (1112, 1090 cm^−1^). The ^1^H NMR spectral data are consistent with literature values and confirm the structure of glabridin.

The naturally low content of glabridin in licorice roots and the presence of structurally related flavonoids significantly complicate its isolation. The applied scheme of sequential extraction and multistage chromatographic purification made it possible to obtain a fraction with a high content of glabridin suitable for pharmaceutical and cosmetic applications.

The extraction of glabridin from licorice root meal demonstrates the possibility of obtaining valuable flavonoids from plant processing waste and confirms the potential of the integrated utilization of licorice raw materials.

### 2.3. Yield and Characterization of 3-Oxoglycyrrhetinic Acid

As a result of recrystallization, 3.9 g of 3-oxoglycyrrhetinic acid was obtained, corresponding to a yield of 0.39% of the air-dried root meal mass.

These ^1^H NMR spectral data correspond to 3-oxoglycyrrhetinic acid (**4**) (Figure 2).

3-oxoglycyrrhetinic acid (**4**). White crystalline powder, melting point 252–254 °C.

IR spectrum (ν, cm^−1^): 2965 (–CH_3_), 1726, 1682, 1643 (C=O), 1141, 1086, 947 (C–O). ^1^H NMR (600 MHz, DMSO-d_6_ + CCl_4_, δ, ppm, *J*/Hz): 1.38 and 2.83 (2H, *m* and *ddd*, 13.3, 7.1, 4.2, H-1), 2.27 and 2.52 (2H, *ddd*, 15.5, 6.6, 4.1 and *ddd*, 15.6, 10.9, 7.2, H-2), 1.32 (1H, *m*, H-5), 1.52 (2H, *m*, H-6), 1.41 and 1.69 (2H, *m* and *ddd*, 12.5, 12.1, 5.6, H-7), 2.39 (1H, *s*, H-9), 5.54 (1H, *s*, H-12), 1.19 and 1.81 (2H, *m* and *m*, H-15), 0.99 and 2.06 (2H, *m* and *ddd*, 13.7, 13.7, 4.4, H-16), 2.18 (1H, *dd*,13.3, 3.7, H-18), 1.56 and 1.82 (2H, *dd*,13.6, 13.4 and *m*, H-19), 1.28 and 1.89 (2H, *m* and *m*, H-21), 1.36 (2H, *m*, H-22), 1.03 (3H, *s*, H-23), 0.99 (3H, *s*, H-24), 1.19 (3H, *s*, H-25), 1.12 (3H, *s*, H-26), 1.37 (3H, *s*, H-27), 0.83 (3H, *s*, H-28), 1.13 (3H, *s*, H-29), 11.95 (*br. s*, OH-30).

The IR spectrum is characterized by intense absorption bands of carbonyl groups (1726, 1682, and 1643 cm^−1^), as well as signals corresponding to C–O bonds (1141 cm^−1^), which are consistent with the structure of an oxo-derivative of a triterpenoid. The ^1^H NMR spectral data agree with literature values and confirm the structure of 3-oxoglycyrrhetinic acid.

3-Oxoglycyrrhetinic acid is a biologically active derivative of glycyrrhetinic acid and is of considerable interest due to its anti-inflammatory, hepatoprotective, and dermatoprotective properties. The isolation of this compound from licorice root meal confirms that the waste products of licorice processing remain a valuable source of triterpenoid compounds.

The efficient extraction of 3-oxoglycyrrhetinic acid after the sequential removal of lipids and flavonoids demonstrates the selectivity of the applied fractionation scheme and its potential for the integrated processing of plant raw materials.

### 2.4. Yield and Characterization of Glycyrrhetinic Acid

As a result of recrystallization, 2.1 g of glycyrrhetinic acid was obtained, corresponding to a yield of 0.21% of the air-dried root meal mass.

These ^1^H NMR spectral data correspond to glycyrrhetinic acid (5) (Figure 2).

Glycyrrhetinic acid (**5**). White crystalline powder, m.p. 295–297 °C.

IR spectrum (ν, cm^−1^): 3435 (OH), 2946, 2861 (–CH_3_, –CH_2_, –CH), 1701, 1662 (C=O), 1177, 1026, 987 (C–O). ^1^H NMR (400 MHz, DMSO-d_6_, δ, ppm, *J*/Hz): 0.96 and 3.24 (2H, *m* and *ddd*,7.8, 9.1, 4.1, H-1), 1.76 and 2.04 (2H, *m* and *m*, H-2), 3.52 (1H, *dd*, 12.4, 5.1, H-3), 0.90 (1H, *m*, H-5), 1.47 (2H, *m*, H-6), 1.49 and 1.76 (2H, *m* and *ddd*, 12.6, 12.1, 5.6, H-7), 2.57 (1H, *s*, H-9), 6.04 (1H, *s*, H-12), 1.26 and 2.17 (2H, *m* and *m*, H-15), 1.05 and 2.18 (2H, *m* and *m*, H-16), 2.20 (1H, *m*, H-18), 1.47 and 2.31 (2H, *m* and *m*, H-19), 1.50 and 2.10 (2H, *m* and *m*, H-21), 1.31 (2H, *m*, H-22), 1.16 (3H, *s*, H-23), 0.83 (3H, *s*, H-24), 1.30 (3H, *s*, H-25), 1.37 (3H, *s*, H-26), 1.38 (3H, *s*, H-27), 1.11 (3H, *s*, H-28), 1.43 (3H, *s*, H-29), 11.90 (*br. s*, OH-30).

The IR spectrum is characterized by absorption bands corresponding to the hydroxyl group (3435 cm^−1^), aliphatic C–H bonds (2946–2861 cm^−1^), carbonyl groups (1701 and 1662 cm^−1^), and C–O bonds (1177–987 cm^−1^). The ^1^H NMR spectral data are consistent with literature values and confirm the structure of glycyrrhetinic acid.

### 2.5. Selection of an Optimal Solvent for Glabridin Extraction

In continuation of the conducted studies, experiments were performed to select an effective solvent for the extraction of the isoflavonoid glabridin and the total flavonoid fraction from licorice root waste with a high yield, as well as to analyze the glabridin content in the obtained extracts using the HPLC method.

For this purpose, licorice root waste (1 kg) was subjected to extraction at room temperature at a (*v*/*v*) 1:10 ratio using methanol, ethanol, and ethyl acetate as solvents. The obtained extracts were filtered separately and concentrated on a vacuum evaporation unit at 45–50 °C to a viscous consistency, followed by drying.

**Figure 3:**
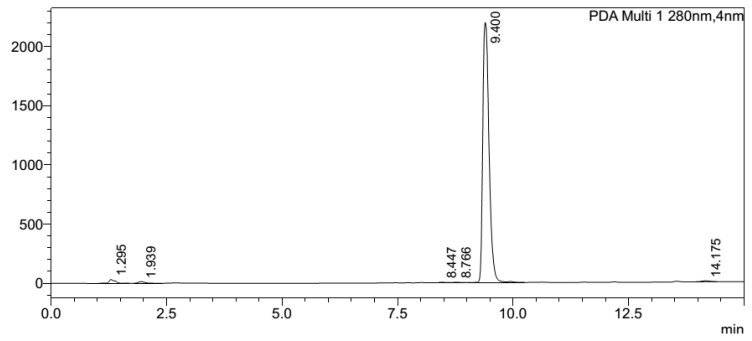
Chromatogram of the standard sample of glabridin. The retention time of glabridin was ~9.4 min.

**Figure 4:**
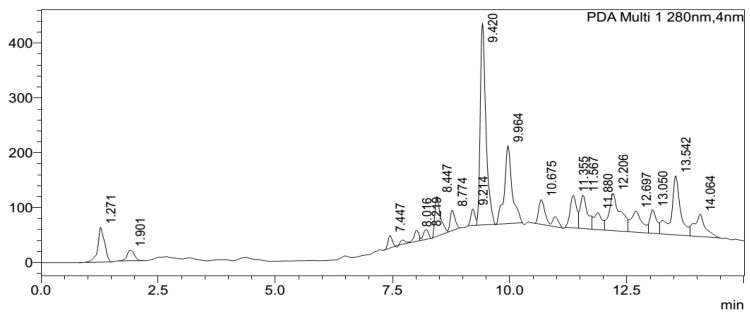
Chromatogram of the methanolic extract of licorice root waste.

**Figure 5:**
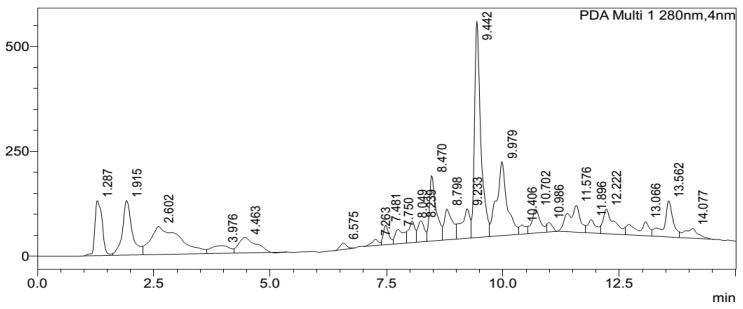
Chromatogram of the ethanolic extract of licorice root waste.

**Figure 6:**
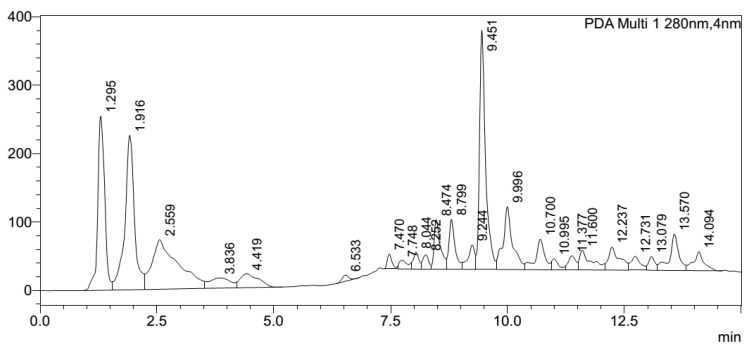
Chromatogram of the ethyl acetate extract of licorice root waste.

Each of the obtained extracts was analyzed qualitatively and quantitatively. The resulting chromatograms are presented in Figure 3, Figure 4, Figure 5 and Figure 6.

Based on the results obtained, a quantitative analysis of the content of glabridin and total flavonoids isolated from licorice root waste using different solvents was carried out. The results of the analysis are presented in Table 2.

All extraction experiments and HPLC analyses were performed in triplicate (*n* = 3), and the results are presented as mean ± standard deviation (SD). Ethanol provided the highest extraction yield (7.10 ± 0.20%), whereas ethyl acetate demonstrated slightly higher selectivity toward glabridin (4.82 ± 0.15%) and total flavonoids (14.80 ± 0.22%).

Values are expressed as mean ± standard deviation (SD) of three independent experiments (*n* = 3). Statistical analysis was performed using one-way ANOVA. Significant differences among solvents were observed for extraction yield (*p*< 0.001) and total flavonoid content (*p* = 0.004), whereas no significant differences were detected for glabridin content (*p* = 0.121).

## 3. Discussion

The detection of β-sitosterol and stigmasterol indicates the presence of biologically active phytosterols in the lipid fraction of licorice root meal. These compounds are known to exhibit anti-inflammatory, membrane-stabilizing, and reparative activities. Their isolation from the waste products of licorice raw material processing confirms the potential of integrated utilization of plant materials and the production of pharmaceutically valuable compounds.

The results demonstrated that the extraction solvent significantly affects both the yield of extractive substances and the content of glabridin and total flavonoids. Ethyl acetate exhibited higher selectivity toward lipophilic compounds, resulting in slightly higher concentrations of glabridin (4.82%) and total flavonoids (14.80%). However, the overall extraction yield obtained with ethyl acetate was lower than that achieved with methanol and ethanol. Ethanol provided the highest extraction yield (7.10%) while maintaining a high concentration of target compounds. Therefore, the selection of ethanol as the optimal extraction solvent was based not only on compound selectivity but also on extraction efficiency, economic feasibility, lower toxicity, environmental safety, ease of solvent recovery, and suitability for large-scale industrial applications. These findings are consistent with recent trends in natural product processing, where ethanol is widely recognized as one of the most promising green solvents for the extraction of plant-derived bioactive compounds due to its renewability, low environmental impact, and compliance with the principles of green chemistry [28,29,30,31].

In recent years, various methods have been proposed for obtaining individual biologically active compounds from licorice roots [32,33], including approaches for the isolation and purification of the aglycone flavonoid glabridin [34,35]. However, technological challenges associated with glabridin recovery are mainly related to its relatively low natural abundance in plant material (0.05–0.23%) and the presence of structurally related flavonoids that complicate separation and purification processes. Consequently, in practical pharmaceutical and cosmetic applications, the production of glabridin-enriched fractions is often considered more feasible and economically justified than the isolation of completely pure glabridin [36,37].

Most previously reported studies have focused on the isolation of individual compounds such as glabridin, glycyrrhizic acid, or glycyrrhetinic acid from licorice roots and extracts. In contrast, the present study proposes a sequential extraction strategy that enables the recovery of lipid fractions, glabridin, 3-oxoglycyrrhetinic acid, and glycyrrhetinic acid from licorice root meal within a single integrated technological scheme. Such an approach improves the utilization efficiency of plant raw materials, increases the value of industrial by-products, and contributes to reducing processing waste through the comprehensive utilization of licorice processing residues. Furthermore, this approach is consistent with modern biorefinery concepts aimed at maximizing the recovery of valuable products from renewable plant biomass and industrial residues [38,39].

Previously proposed approaches for processing licorice root meal have allowed the additional recovery of certain groups of compounds, such as total flavonoids (4.66%) and free glycyrrhizic acid (0.91%) [40,41,42]. However, these methods are generally fragmentary and do not ensure the comprehensive recovery of all valuable constituents present in the plant residue.

The sequential extraction of lipids, glabridin, and triterpenoid aglycones demonstrated in the present study indicates the high efficiency of the proposed method for the comprehensive processing of *G. glabra* root meal. Compared with conventional technologies aimed primarily at obtaining water-soluble compounds, the proposed approach provides several advantages. It enables a more complete recovery of lipophilic biologically active substances, expands the spectrum of pharmacologically valuable compounds obtained from licorice raw materials, allows the utilization of root meal as a secondary plant resource, and contributes to reducing the amount of industrial processing waste. Overall, the results demonstrate that licorice root meal should be considered not as a processing waste but as a valuable secondary raw material containing multiple classes of biologically active compounds suitable for pharmaceutical and cosmetic applications.

## 4. Materials and Methods

### 4.1. Plant Material

Licorice (*G. glabra* L.) root meal obtained after industrial extraction of water-soluble constituents was used as the plant material in this study. The raw material was air-dried, ground to an appropriate particle size, and stored in a dry and well-ventilated place until further experimental procedures.

### 4.2. Chemicals and Reagents

Ethanol (95%), petroleum ether, ethyl acetate, and other solvents of analytical grade were used in the experiments. Silica gel (KSK grade, Lumex, Saint Petersburg, Russia) was employed for column chromatography, while Sephadex^®^ LH-20 (GE Healthcare, Waukesha, WI, USA) was used for final purification of the isolated compounds. Standard samples of phytosterols were applied for comparative analysis during thin-layer chromatography.

### 4.3. Analytical Methods

Thin-layer chromatography (TLC) was performed on Merck silica gel plates (Darmstadt, Germany) (254 nm) using petroleum ether–ethyl acetate solvent systems. Quantitative determination of glabridin and purity assessment of the isolated fractions were carried out using high-performance liquid chromatography (HPLC).

HPLC analysis was performed on a Shimadzu LC-20 (Kyoto, Japan) chromatographic system equipped with a UV detector and autosampler. Separation was achieved on a Supelco C18 column (Merck Group, Germany) (150 × 4.6 mm, 5 μm particle size). The mobile phase consisted of solvent A (0.1% aqueous formic acid) and solvent B (acetonitrile). Elution was performed under isocratic conditions throughout the analysis. The flow rate was 1.0 mL min^−1^, the column temperature was maintained at 40 °C, the injection volume was 20 μL, and detection was carried out at 282 nm. Under these conditions, the retention time of glabridin was approximately 9.4 min.

Quantification was performed using an external standard method. A reference standard of glabridin (Sigma-Aldrich, St. Louis, MO, USA, purity 98%, SKU G9548-5MG) was used for calibration. Calibration solutions were prepared within the concentration range of 5–50 μg mL^−1^, and the calibration curve demonstrated excellent linearity (R^2^ = 0.9997).

### 4.4. Preparation of Ethanol Extract

Air-dried root meal of *G. glabra* L. (1.0 kg), obtained after removal of water-soluble components, was placed in an extractor and subjected to maceration with 95% ethanol. The solvent was added until complete immersion of the plant material. The first extraction was carried out using 7 L of ethanol. Extraction was performed four times with a raw material–solvent ratio of *w*/*w* 1:7. Each extraction stage lasted 8 h. The combined extracts were concentrated under reduced pressure to obtain a dry residue (77.6 g).

### 4.5. Isolation of Lipid Fraction

The obtained dry residue was successively treated with petroleum ether–ethyl acetate (*v*/*v* 9:1) three times using 200 mL portions of solvent. The combined extracts were evaporated to dryness to obtain the lipid fraction.

The completeness of lipid extraction was monitored by TLC using standard samples of sterols and fatty acids. Phytosterols present in the lipid fraction were further isolated by column chromatography on silica gel (KSK grade) using petroleum ether–ethyl acetate (*v*/*v* 9.5:0.5) as the eluent. The structures of the isolated compounds were determined by ^1^H and ^13^C NMR spectroscopy.

### 4.6. Isolation of Glabridin

The residue of the ethanol extract after removal of the lipid fraction was treated three times with petroleum ether–ethyl acetate (*v*/*v* 4:1) using 200 mL portions of solvent. The combined extracts were concentrated under reduced pressure to obtain a fraction containing glabridin (37 g). To remove residual lipids, the obtained fraction was additionally treated once with petroleum ether–ethyl acetate (*v*/*v* 9:1). The solution was separated by decantation.

The remaining residue (36 g) was mixed with silica gel (1:1) and applied onto a column packed with silica gel (1:2). Elution was performed using petroleum ether–ethyl acetate (6:1), yielding a fraction (5.7 g) containing 49% glabridin.

This fraction was further purified by repeated column chromatography on silica gel (*v*/*v* 1:2) using petroleum ether–ethyl acetate (*v*/*v* 5:1) as the eluent, producing a fraction (2.8 g) containing 69.8% glabridin. Final purification was performed on a Sephadex^®^ LH-20 column using ethanol as the eluent, yielding 1.95 g of a fraction containing glabridin with a purity of 87.9%. The yield of glabridin was 0.17% relative to the air-dried root meal. The structure of the compound was confirmed using IR and ^1^H NMR spectroscopy.

### 4.7. Isolation of 3-Oxoglycyrrhetinic Acid

The residue remaining after removal of the lipid fraction and glabridin was treated three times with petroleum ether–ethyl acetate (*v*/*v* 3:1) using 200 mL portions of solvent. The combined extracts were concentrated under reduced pressure to obtain a fraction containing 3-oxoglycyrrhetinic acid. The obtained fraction (5.2 g) was purified by recrystallization from 85% aqueous ethanol to yield pure 3-oxoglycyrrhetinic acid. The structure of the compound was confirmed by IR and ^1^H NMR spectroscopy.

### 4.8. Isolation of Glycyrrhetinic Acid

**Figure 7:**
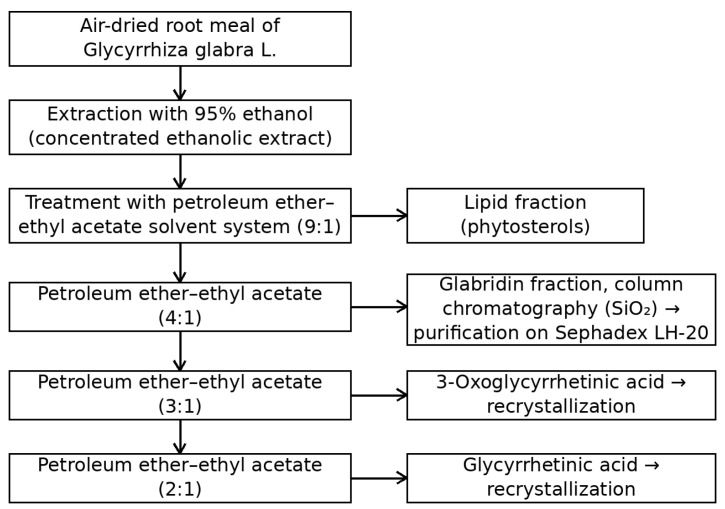
General scheme for the isolation of hydrophobic biologically active compounds from the root meal of *Glycyrrhiza glabra* L.

The residue remaining after removal of lipids, glabridin, and 3-oxoglycyrrhetinic acid was extracted three times with petroleum ether–ethyl acetate (2:1, *v*/*v*) using 200 mL portions of solvent. The combined extracts were concentrated under reduced pressure to obtain a fraction containing glycyrrhetinic acid. The obtained fraction (2.8 g) was purified by recrystallization from 80% aqueous ethanol to yield pure glycyrrhetinic acid. The structure of the compound was confirmed using IR and ^1^H NMR spectroscopy. The completeness of extraction of glabridin, 3-oxoglycyrrhetinic acid, and glycyrrhetinic acid was monitored by TLC on Merck plates (254 nm) using a benzene–ethyl acetate (2:1) solvent system, as well as by HPLC analysis (Figure 7).

### 4.9. Statistical Analysis

All extraction experiments and HPLC analyses were performed in triplicate (*n* = 3). Results are expressed as mean ± standard deviation (SD). Statistical evaluation of differences among extraction solvents was performed using one-way analysis of variance (ANOVA). Statistical significance was accepted at *p* < 0.05.

## 5. Conclusions

In the present study, an efficient approach for the comprehensive recovery of hydrophobic biologically active compounds from the root meal of *G. glabra*, obtained after the removal of water-soluble constituents, was developed. Sequential ethanol extraction followed by selective fractionation using a petroleum ether–ethyl acetate solvent system and chromatographic purification enabled the isolation of several valuable lipophilic compounds. As a result, a lipid fraction containing phytosterols (β-sitosterol and stigmasterol), the aglycone flavonoid glabridin, and triterpenoid aglycones including 3-oxoglycyrrhetinic acid and glycyrrhetinic acid were successfully isolated and characterized.

Comparative solvent studies demonstrated that ethanol provided the highest extraction yield while maintaining high levels of glabridin and total flavonoids, supporting its suitability as an extraction solvent for the comprehensive processing of licorice root meal. The proposed extraction scheme provides a more efficient recovery of lipophilic compounds compared with conventional processing technologies focused primarily on water-soluble constituents.

The results demonstrate that licorice root meal represents a valuable secondary plant resource for the recovery of biologically active substances. Comprehensive processing of this industrial by-product contributes to the rational utilization of plant resources, reduction of processing waste, and improvement of the overall economic efficiency of licorice-processing technologies.

Future studies will focus on improving the purity of glabridin-containing fractions, evaluating the biological activities of the isolated compounds and fractions, and scaling up the proposed extraction process for pilot-scale and industrial applications.

## Figures and Tables

**Table 1:** Yield and purity of hydrophobic compounds isolated from the root meal of *G. glabra* L.

Compound	Yield, % (*w*/*w*)	Purity, %	Analytical Confirmation	Notes
Lipid fraction	1.1	—	TLC	mixture of neutral lipids
β-Sitosterol	n.d. *	>95	^1^H, ^13^C NMR; TLC	phytosterol
Stigmasterol	n.d. *	>95	^1^H, ^13^C NMR; TLC	phytosterol
Glabridin	0.17	87.9	NMR, IR, HPLC	flavonoid aglycone
3-Oxoglycyrrhetinic acid	0.39	≈98	NMR, IR	triterpenoid
Glycyrrhetinic acid	0.21	≈98	NMR, IR	aglycone of glycyrrhizin

* n.d.—not determined individually (isolated as part of the lipid fraction).

**Table 2:** Comparison of extraction yield, glabridin content, and total flavonoid content in extracts obtained from licorice root meal using different solvents.

No.	Solvent	Yield of Extractive Substances %	Glabridin Content in Extractives %	Total Flavonoid Content in Extractives %
1	Methanol	6.60 ± 0.08	4.75 ± 0.04	14.50 ± 0.07
2	Ethanol	7.10 ± 0.20	4.65 ± 0.20	14.45 ± 0.28
3	Ethyl acetate	5.40 ± 0.14	4.82 ± 0.15	14.80 ± 0.22

## Data Availability

The data presented in this study are available in the article.

## Abbreviations

The following abbreviations are used in this manuscript:

TLC	Thin-layer chromatography
HPLC	High-performance liquid chromatography
IR	Infrared spectroscopy
NMR	Nuclear magnetic resonance
*v*/*v*	Volume per volume
*w*/*w*	Weight per weight

## References

[1] Bhan M., Satija S., Garg C., Dureja H., Garg M. (2017). A novel approach towards green extraction for glycyrrhetinic acid by ionic liquid-based microwave-assisted extraction and optimization through response surface methodology. Pharmacogn. J..

[2] Hayashi H., Fukui H., Tabata M. (2003). Field survey of *Glycyrrhiza* plants in Central Asia (3): Chemical characterization of *Glycyrrhiza glabra* collected in Uzbekistan. Chem. Pharm. Bull..

[3] Ammosov A.S., Litvinenko V.I. (2003). Triterpenoids of plants of the genera *Glycyrrhiza* L. and *Meristotropis Fisch. et Mey* genuses (A review). Pharm. Chem. J..

[4] Damle M. (2014). *Glycyrrhiza glabra* (Licorice)—A potent medicinal herb. Int. J. Herb. Med..

[5] Alwan A.M., Nesrullah Z., Faraj E. (2015). Study on the effect of ethanolic extract of *Glycyrrhiza glabra* on pathogenic bacteria. Int. J. Curr. Microbiol. Appl. Sci..

[6] Simmler C., Pauli G.F., Chen S.N. (2013). Phytochemistry and biological properties of glabridin. Fitoterapia.

[7] Pastorino G., Cornara L., Soares S., Rodrigues F., Oliveira M.B.P.P. (2018). Liquorice (*Glycyrrhiza glabra*): A phytochemical and pharmacological review. Phytother. Res..

[8] Graebin C.S. (2018). The pharmacological activities of glycyrrhizic acid and glycyrrhetinic acid. Reference Series in Phytochemistry.

[9] Ammosov A.S., Litvinenko V.I. (2007). Phenolic compounds of the genera *Glycyrrhiza* L. and *Meristotropis Fisch. et Mey* (Review). Pharm. Chem. J..

[10] Ain N.U., Wu S., Li X., Li D., Zhang Z. (2022). Isolation, Characterization, Pharmacology and Biopolymer Applications of Licorice Polysaccharides: Review. Materials.

[11] Nascimento M.H.M., de Araújo D.R. (2022). Exploring the pharmacological potential of glycyrrhizic acid: From therapeutic applications to trends in nanomedicine. Future Pharmacol..

[12] Taarji N., Bouhoute M., Fainassi F., Hafidi A., Kobayashi I., Neves M.A., Tominaga K., Isoda H., Nakajima M. (2021). Interfacial and emulsifying properties of purified glycyrrhizin and non-purified glycyrrhizin-rich extracts from liquorice root (*Glycyrrhiza glabra*). Food Chem..

[13] Kagueyam S.S., dos Santos Filho J.R., Contato A.G., de Souza C.G.M., Castoldi R., Corrêa R.C.G., Conte Junior C.A., Yamaguchi N.U., Bracht A., Peralta R.M. (2025). Green Extraction of Bioactive Compounds from Plant-Based Agri-Food Residues: Advances toward Sustainable Valorization. Plants.

[14] Zaky A.A., Akram M.U., Rybak K., Witrowa-Rajchert D., Nowacka M. (2024). Bioactive Compounds from Plants and By-Products: Novel Extraction Methods, Applications, and Limitations. AIMS Mol. Sci..

[15] Bekavac N., Krog K., Stanić A., Šamec D., Šalić A., Benković M., Jurina T., Gajdoš Kljusurić J., Valinger D., Jurinjak Tušek A. (2025). Valorization of Food Waste: Extracting Bioactive Compounds through Sustainable Technologies. Antioxidants.

[16] Chaachouay N., Zidane L. (2024). Plant-Derived Natural Products: A Source for Drug Discovery and Development. Drugs Drug Candidates.

[17] El-Saadony M.T., Saad A.M., Mohammed D.M., Korma S.A., Alshahrani M.Y., Ahmed A.E., Ibrahim E.H., Salem H.M., Alkafaas S.S., Saif A.M. (2025). Medicinal Plants: Bioactive Compounds, Biological Activities and Therapeutic Applications. Front. Immunol..

[18] Latif R., Nawaz T. (2026). Medicinal Plants and Human Health: A Comprehensive Review of Bioactive Compounds, Therapeutic Effects, and Applications. Phytochem. Rev..

[19] Juárez-Robles E.I., Barrón-Velázquez R., Macías-Alonso M., Hernández-Soto R., Córdova-Guerrero I., Marrero J.G. (2026). Recovery of Bioactive Plant Compounds from Biomass Waste Using Sustainable Methods: A Review. RSC Sustain..

[20] Hao D.-C., He C.-N., Spjut R.W., Xiao P.-G. (2022). Editorial: Plant-Derived Natural Compounds in Drug Discovery: The Prism Perspective between Plant Phylogeny, Chemical Composition, and Medicinal Efficacy. Front. Plant Sci..

[21] Abdurakhmanov B.A., Sotimov G.B., Khalilov R.M., Mamatkhanov A.U. (2021). Technology for Obtaining a Flavonoid-Based Substance from the Aerial Parts of *Glycyrrhiza glabra*. Pharm. Chem. J..

[22] Khaled S.M. (2017). Composition and Properties of Biologically Active Substances of *Glycyrrhizae radices*. Ph.D. Thesis.

[23] Chen X., Zhang Y., Liu Q. (2024). Green extraction technologies for recovery of bioactive compounds from plant waste. Green Chem..

[24] Khabibrakhmanova V.R., Khaled S.M., Gabdrakhmanova A.R., Sysoeva M.A. (2016). Processing of Licorice Root Meal. II. Triterpenoid and Flavonoid Compounds of Ethanolic Extracts. Chem. Plant Raw Mater..

[25] (2004). Method for Extracting Glycyrrhizic Acid from Licorice.

[26] (2016). Method for Extracting and Purifying Glabridin from Glycyrrhiza glabra Residues.

[27] Lim T.K. (2016). Edible Medicinal and Non-Medicinal Plants; Modified Stems, Roots, Bulbs.

[28] Chemat F., Abert-Vian M., Ravi H.K., Khadhraoui B., Hilali S., Perino S., Fabiano-Tixier A.S. (2019). Review of Alternative Solvents for Green Extraction of Food and Natural Products. Molecules.

[29] Martins R., Barbosa A., Advinha B., Nunes M.C., Gonçalves A.C., Alves M.J., Oliveira M.B.P.P., Silva L.R. (2023). Green Extraction Techniques of Bioactive Compounds: A State-of-the-Art Review. Processes.

[30] Chemat F., Vian M.A., Cravotto G. (2012). Green Extraction of Natural Products: Concept and Principles. Int. J. Mol. Sci..

[31] Clark J.H., Deswarte F.E.I., Farmer T.J. (2009). The Integration of Green Chemistry into Future Biorefineries. Biofuels Bioprod. Biorefin..

[32] Denisova S.B., Danilov V.T., Yunusova S.G., Davydova V.A., Murinov Y.I., Zarudiy F.S. (2007). Isolation and Biological Activity of Lipids from Roots of *Glycyrrhiza glabra*. Pharm. Chem. J..

[33] Hasan M.K., Ara I., Mondal M.S.A., Kabir Y. (2021). Phytochemistry, pharmacological activity and potential health benefits of *Glycyrrhiza glabra*. Heliyon.

[34] Tian M., Yan H. (2008). Extraction of glycyrrhizic acid and glabridin from licorice. Int. J. Mol. Sci..

[35] Xu Y., Yuan Q., Hou X. (2009). Preparative separation of glabridin from *Glycyrrhiza glabra* extracts with microporous resins. Sep. Sci. Technol..

[36] Ma S., Muhebuli A., Bahargul K., He Q. (2007). Preparation process of glabridin, an isoflavone component of licorice root. Xinjiang Med. Univ. J..

[37] Karkanis A., Martins N., Petropoulos S., Ferreira I.C.F.R. (2018). Phytochemical composition, health effects, and crop management of liquorice (*Glycyrrhiza glabra* L.): A medicinal plant. Food Rev. Int..

[38] Wang L., Yang R., Yuan B., Liu Y., Liu C. (2015). The antiviral and antimicrobial activities of licorice, a widely used Chinese herb. Acta Pharm. Sin. B.

[39] Dong W., Guo J., Wen H., Liu D., Xia K. (2010). Separation and Purification Method of High-Purity Glabridin.

[40] Liuhua S. (2011). Method for Preparing Glabridin.

[41] Yu X., Lin S., Zhou Z., Chen X., Liang J., Yu X., Chowbay B., Wen J.Y., Duan W., Chan E. (2007). Role of P-glycoprotein in Limiting the Brain Penetration of Glabridin, an Active Isoflavan from the Root of *Glycyrrhiza glabra*. Pharm. Res..

[42] Hu C. (2010). Method for Producing High-Purity Glabridin.

